# Developing and evaluating Birthing on Country services for First Nations Australians: the Building On Our Strengths (BOOSt) prospective mixed methods birth cohort study protocol

**DOI:** 10.1186/s12884-022-05277-8

**Published:** 2023-01-28

**Authors:** Penny Haora, Yvette Roe, Sophie Hickey, Yu Gao, Carmel Nelson, Jyai Allen, Melanie Briggs, Faye Worner, Sue Kruske, Kristie Watego, Sarah-Jade Maidment, Donna Hartz, Juanita Sherwood, Lesley Barclay, Sally Tracy, Mark Tracy, Liz Wilkes, Roianne West, Nerida Grant, Sue Kildea

**Affiliations:** 1 Molly Wardaguga Research Centre, Charles Darwin University, Ann Street, Brisbane, QLD 4000 Australia; 2 Waminda South Coast Women’s Health & Wellbeing Aboriginal Corporation, Kinghorne Street, Nowra, NSW 2541 Australia; 3 Molly Wardaguga Research Centre, Charles Darwin University, Darwin, Australia; 4 Institute for Urban Indigenous Health, Cox Road, Windsor, QLD 4030 Australia; 5 Poche Centre for Indigenous Health, University of Queensland, Brisbane, QLD Australia; 6 Molly Wardaguga Research Centre, Charles Darwin University, Grevillea Drive, Sadadeen, NT 0870 Australia; 7 The University of Sydney, Camperdown, NSW 2006 Australia; 8 My Midwives Brisbane, Windsor Road, Red Hill, QLD 4059 Australia; 9 Congress of Aboriginal & Torres Strait Islander Nurses and Midwives, Lytton Road, Murarrie, QLD 4172 Australia

**Keywords:** Midwifery, Health services research, Aboriginal and Torres Strait islander, Indigenous, First Nations, Health disparities, Birthing on Country, Prospective birth cohort, Preterm birth

## Abstract

**Background:**

With the impact of over two centuries of colonisation in Australia, First Nations families experience a disproportionate burden of adverse pregnancy and birthing outcomes. First Nations mothers are 3–5 times more likely than other mothers to experience maternal mortality; babies are 2–3 times more likely to be born preterm, low birth weight or not to survive their first year. ‘Birthing on Country’ incorporates a multiplicity of interpretations but conveys a resumption of maternity services in First Nations Communities with Community governance for the best start to life. Redesigned services offer women and families integrated, holistic care, including carer continuity from primary through tertiary services; services coordination and quality care including safe and supportive spaces. The overall aim of Building On Our Strengths (BOOSt) is to facilitate and assess Birthing on Country expansion into two settings - urban and rural; with scale-up to include First Nations-operated birth centres. This study will build on our team’s earlier work - a Birthing on Country service established and evaluated in an urban setting, that reported significant perinatal (and organisational) benefits, including a 37% reduction in preterm births, among other improvements.

**Methods:**

Using community-based, participatory action research, we will collaborate to develop, implement and evaluate new Birthing on Country care models. We will conduct a mixed-methods, prospective birth cohort study in two settings, comparing outcomes for women having First Nations babies with historical controls. Our analysis of feasibility, acceptability, clinical and cultural safety, effectiveness and cost, will use data including (i) women’s experiences collected through longitudinal surveys (three timepoints) and yarning interviews; (ii) clinical records; (iii) staff and stakeholder views and experiences; (iv) field notes and meeting minutes; and (v) costs data. The study includes a process, impact and outcome evaluation of this complex health services innovation.

**Discussion:**

Birthing on Country applies First Nations governance and cultural safety strategies to support optimum maternal, infant, and family health and wellbeing. Women’s experiences, perinatal outcomes, costs and other operational implications will be reported for Communities, service providers, policy advisors, and for future scale-up.

**Trial registration:**

Australia & New Zealand Clinical Trial Registry #ACTRN12620000874910 (2 September 2020).

**Supplementary Information:**

The online version contains supplementary material available at 10.1186/s12884-022-05277-8.

## Background

With more than 200 years of colonisation in Australia, maternal and infant health disparities are frequently reported for First Nations families compared with non-First Nations [1]. The maternal mortality rate is between 3 and 5 times higher for First Nations women, and other perinatal indicators show stark discrepancies, including preterm birth (14% versus 8%), low birth weight (12% versus 6%) and perinatal deaths (12 versus 9 per 1000 births) [2].

While perinatal mortality is relatively rare in Australia overall, preterm birth and low birth weight are much more common. Reducing preterm births is an international priority, since it is associated with factors influencing poor health in the short- and long-terms, including adult chronic diseases [3]. Many factors associated with preterm birth are also considered modifiable. Known risk factors include maternal psychosocial stress [4], antenatal infections [5], cigarette smoking in pregnancy [6] and exposure to environmental tobacco [7], low/high body mass index [8], lower maternal education and young maternal age [9] among others.

### Birthing on Country

Over many decades, First Nations communities within Australia have sought to reclaim birth, endeavouring to ensure the best start to life for future generations [10–14]. Following a review of maternity services [15], the Australian Health Ministers Advisory Council (AHMAC), in the National Maternity Services plan, outlined three priority areas for improving First Nations maternity care [16]. First, develop Birthing on Country as the gold standard maternity service model; second, develop and support a First Nations maternity workforce; and third, develop and expand culturally competent maternity care. Subsequently, the Australian government commissioned a review of international literature concerning Birthing on Country. Essential components of Birthing on Country services were identified in the review [17], and were confirmed during a national Birthing on Country workshop [12] (see Table 1). Birthing on Country services were defined (p. 5) as:

*‘maternity services designed and delivered for Indigenous women that encompass some or all of the following elements: are community based and governed; allow for incorporation of traditional practice; involve a connection with land and country; incorporate a holistic definition of health; value Indigenous and non-Indigenous ways of knowing and learning, risk assessment and service delivery; are culturally competent and are developed by, or with, Indigenous people’* [17]*.*

Crucially, First Nations stakeholders at the workshop asserted that Birthing on Country could only be successful through whole system transformation. Participants stated (p. 33) Birthing on Country is about ‘…being able to have babies safely on Country’ [12], and ‘(it) deals with socio-cultural and spiritual risk …not dealt with in the current [maternity care] systems’ (p. 24).

**Table 1:** Key components of exemplary Birthing on Country (BoC) services and differences between standard care (control) and BoC model (new service - complex innovation)

Differences between care models^a^		
	Standard Maternity Care (Control Group)	Birthing on Country service (Innovation Group)
Governance	No First Nations governance mechanism that actively engages local Community and supports oversight of maternity services and outcomes.	First Nations governance of the service (ownership through governance committee with oversight mechanisms).
Workforce strategy	No specific pathways to support First Nations MIH workforce.	Supportive career pathways with First Nations MIH workers embedded in the team with cadetships (or similar) to support midwifery education for First Nations persons. Aboriginal Family Support Workers (or similar) work side-by-side with midwives.
Continuity	No continuity of midwifery carer through antenatal, intrapartum and postnatal continuum. Care is fragmented and women see multiple providers.	Continuity of midwifery carer networked from community to a higher-level service, offering 24/7 care from a named midwife from first presentation in pregnancy until handover to child health services 6 weeks after birth. Midwifery Group Practice (caseload) midwife providing clinical care in collaboration with the extended workforce based on need (e.g. psychologist, social worker, obstetrician, paediatricians, sexual and reproductive health).
Strengthening family capacity	No maternity programs focussed on strengthening family capacity and/or cultural connection in relation to services offered through this hospital. Women may enrol in external programs.	Programs that focus on strengthening family capacity and cultural connection in relation to MIH, integrated with the BoC service.
Place of birth	No out-of-hospital option. Hospitals do not recognise or incorporate the knowledge, values and birth aspirations of First Nations people.	During the first and second phase of the new care model, all intrapartum care will occur in hospitals. If feasible, a free-standing, community-based birth centre will be set up and available for First Nations women with uncomplicated pregnancies (Category A according to consultation and referral guidelines) [18]; women with complex pregnancies (Category B or C) will birth in the local or tertiary hospital. Birth care will incorporate traditional practice; recognise the connection with Land and Country; incorporate holistic health; value both First Nations and non-First Nations ways of knowing, learning and risk assessment; and will provide a culturally competent service.

Note: *MIH* Maternal and Infant Health

^a^Some sections reproduced from Kildea, Gao et al. [19] Permission not required under licence CC BY-NC-ND 4.0

Guiding principles for developing Birthing on Country services and an evaluation framework (The Framework) were endorsed by the Australian Health Ministers Advisory Council in 2016 [20]. ‘Exemplar sites’ that return birthing services to First Nations Community control in urban, rural, remote and very remote locations were recommended [12]. Despite this, there is no evidence of action, implementation or intention from within the mainstream maternity care system. To date, only one such exemplar has been developed and evaluated in urban Australia, with improved outcomes for mothers and babies across multiple indicators [21]. The Building On Our Strengths (BOOSt) study will apply the guiding principles to support successful service model development and will extend the evaluation framework by developing a more comprehensive evaluation system.

### The Birthing in Our Community (BiOC) service

The Birthing in Our Community (BiOC) Service was established through partnership between two First Nations Community Controlled Health Organisations and a mainstream maternity service provider in an urban Australia setting. The service aimed to reform and reorient systems to respond to the needs and aspirations of First Nations women and families and in doing so, to address known disparities in birth outcomes. First Nations leadership in design and implementation of this new service was pivotal. The Indigenous Birthing in an Urban Setting (IBUS) study supported this service reorientation and used a participatory action research (PAR) approach to investigate BiOC’s implementation and impact [11]. IBUS reported: significantly lower rates of preterm birth compared with standard care [19, 22]; that mothers were more likely to attend five or more antenatal care (ANC) visits and to be exclusively breastfeeding at hospital discharge [19]. Besides the important clinical outcomes assessed, IBUS used multiple methods to assess the broader social and wellbeing status of mothers as they journeyed through the new Birthing on Country service; and undertook an economic evaluation (forthcoming). IBUS was the first published evaluation describing the development, outcomes and effectiveness of implementing Birthing on Country policy in Australia [19]. Consequently, BOOSt privileges the aspirations of First Nations Elders and Communities to redesign maternity care to address systemic, structural inequities affecting the health and wellbeing of mothers, babies and families [16].

### Community-based Birth Centres & Carer Continuity

First Nations birth centres are an important component of the ‘full’ implementation of Birthing on Country services. Such birth centres are aligned with a desire, noted internationally, for birthing sovereignty, and the need for culturally and clinically safe care and birth places [23, 24]. Also known as primary maternity units, birth centres are an important strategy for returning birthing to rural and remote locations. Birth centres emphasise holistic ‘social’ and/or ‘physiological’ birth, therefore, are in demand in any location, and there is strong empirical evidence that they provide safe perinatal care for ‘low-risk’ women [23–30]. Large studies have found no differences in perinatal mortality for women and infants utilising birth centres compared with standard maternity care locations [25, 31, 32]. Additionally, there were better outcomes or no differences related to perinatal morbidity [25, 31–34]; improved outcomes for maternal morbidity [26, 35]; improved outcomes for birth interventions (e.g. fewer caesarean sections) [25, 26, 32, 34, 35] and improved neonatal outcomes [34, 36].

Sound evidence indicates the need for urgent reform of fragmented maternity care, and various plans and strategies aspire to achieve this [37]. Findings from a Cochrane review indicated outcomes for women and babies were significantly improved when care was offered by a known and trusted midwife, usually organised through a midwifery group practice (MGP) [38]. Outcomes included reductions in: preterm birth, fetal loss, early neonatal death, regional analgesia, and instrumental birth. Increases in spontaneous vaginal birth were also found. In Australia, the M@NGO trial [39] confirmed the safety and effectiveness of caseload care (one-to-one known midwife) for all-risk women. However, few MGPs internationally or in Australia focus on First Nations women [13] despite continuity of care and carer having been identified as an important component of culturally safe care for First Nations women globally [40]. In some parts of Australia, there are targeted maternity service models for First Nations women, but continuity does not extend beyond pre and postnatal care.

## Methods

Table 1 articulates the Birthing on Country services innovation to be tested during the BOOSt study, and differentiates this from standard care.

### Study aims, objectives and hypothesis

The BOOSt study aims to determine the:

- feasibility of establishing Birthing on Country services, inclusive of birth centres, in urban and rural settings
- acceptability of the new Birthing on Country services for women and families, Communities and health service providers
- clinical and cultural safety of the new services
- clinical and cost-effectiveness,
- key processes required to establish Birthing on Country birth centres and to create sustainability.

The primary hypothesis is that maternity care from a Birthing on Country service will improve maternal and newborn outcomes for First Nations mothers and babies, when compared to historical controls and a standard care cohort (where available).

### Study design

BOOSt is a participatory action research (PAR) study, using mixed methods to develop, implement and evaluate new Birthing on Country services with free-standing birth centres, in urban and rural settings. While BOOSt is a collaboration [41] between First Nations and non-First Nations partner organisations, our approach privileges First Nations knowledges, research methodologies [42, 43] and ways of knowing, being and doing; based on the following four principles [44]:

1. Value First Nations worldviews, knowledges and realities
2. Honour cultural protocols
3. Emphasise social, historical and political contexts
4. Privilege First Nations voices and experiences.

The PAR design applies a transformative/advocacy lens which drives a social justice agenda [41] (i.e. equity in health access, autonomy, improved outcomes and experiences for First Nations families). The process is dynamic and cyclical, using multiple data sources to identify and analyse issues, plan and implement actions and potential solutions, continuously evaluate and interpret impact, and revise in response to the evaluation [41]. Since PAR engages partners, locally-led co-creation of the new services is assured, with First Nations ways of knowing, being and doing taking precedence, and ensuring the relevance of evidence and knowledge generated [45].

BOOSt consists of multiple study components, including a prospective cohort study, and analyses multiple outcomes, employing multiple data sources to do so. BOOSt includes quantitative and qualitative research components to meet its aims.

### Study settings & populations

BOOSt is funded for 5 years (2018–2022),^1^ and will occur in two sites based around current and planned birthing service catchment areas. BOOSt is based on a collaboration with three Aboriginal Community Controlled Health Organisations (ACCHOs), several peak professional organisations, and numerous health services (and management) organisations (see for more information Additional File 1: BOOSt Study Partner Organisations and their Roles).

#### *Study site 1 urban: Brisbane (otherwise known as* Meanjin*) north, Queensland*

In the urban North Brisbane site, the BOOSt partners are the Institute for Urban Indigenous Health (IUIH), the Moreton Aboriginal and Torres Strait Islander Community Health Service (MATSICHS), and My Midwives (a private midwifery organisation). The new service will cater to the large and rapidly growing First Nations population in the area [46]. My Midwives have had visiting access agreements^2^ with three maternity hospitals in the region for 7 years and they are partnering with IUIH to provide services for First Nations women and families. IUIH and Aboriginal and Torres Strait Islander Community Health Services (ATSICHS) Brisbane Limited are First Nations Community Controlled Health Organisations and both were key partners in development of the first Birthing on Country service (BiOC) in South Brisbane, the forerunner to BOOSt [22, 47].

#### Study site 2 rural: Nowra, New South Wales (NSW)

The rural site is in Nowra (Shoalhaven District NSW, on the unceded Lands of the Yuin Nation) and the key partner is Waminda South Coast Women’s Health and Welfare Aboriginal Corporation (hereby respectfully referred to as Waminda). While centred in the Shoalhaven Local Government Area Waminda’s catchment extends to the Illawarra in the north, west into the Southern Highlands, and to the Far South Coast, where over 22,200 people identifying as First Nations reportedly lived in 2021 [48]. The Shoalhaven area contains a large First Nations population, where First Nations people reported a median weekly income comparatively lower to that of the surrounding areas [48]. In 2018, there were more than 453 First Nations babies born in the Illawarra Shoalhaven area. Births have increased by approximately 30% within 6 years and are expected to continue growing as ~ 35% of the First Nations population is under 15 years and increasing numbers of young women are entering childbearing age [49]. Waminda has a long-held vision to reclaim birth, enable Birthing on Country, and to operate a purpose-built Aboriginal birth centre on the Lands of the Yuin Nation. BOOSt will support Waminda staff to provide birthing services in addition to existing antenatal and postnatal care. The first phase will be implementation of a caseload midwifery group practice providing intrapartum care in the hospital, a collaboration with Shoalhaven District Memorial Hospital (SDMH).

**Fig. 1:**
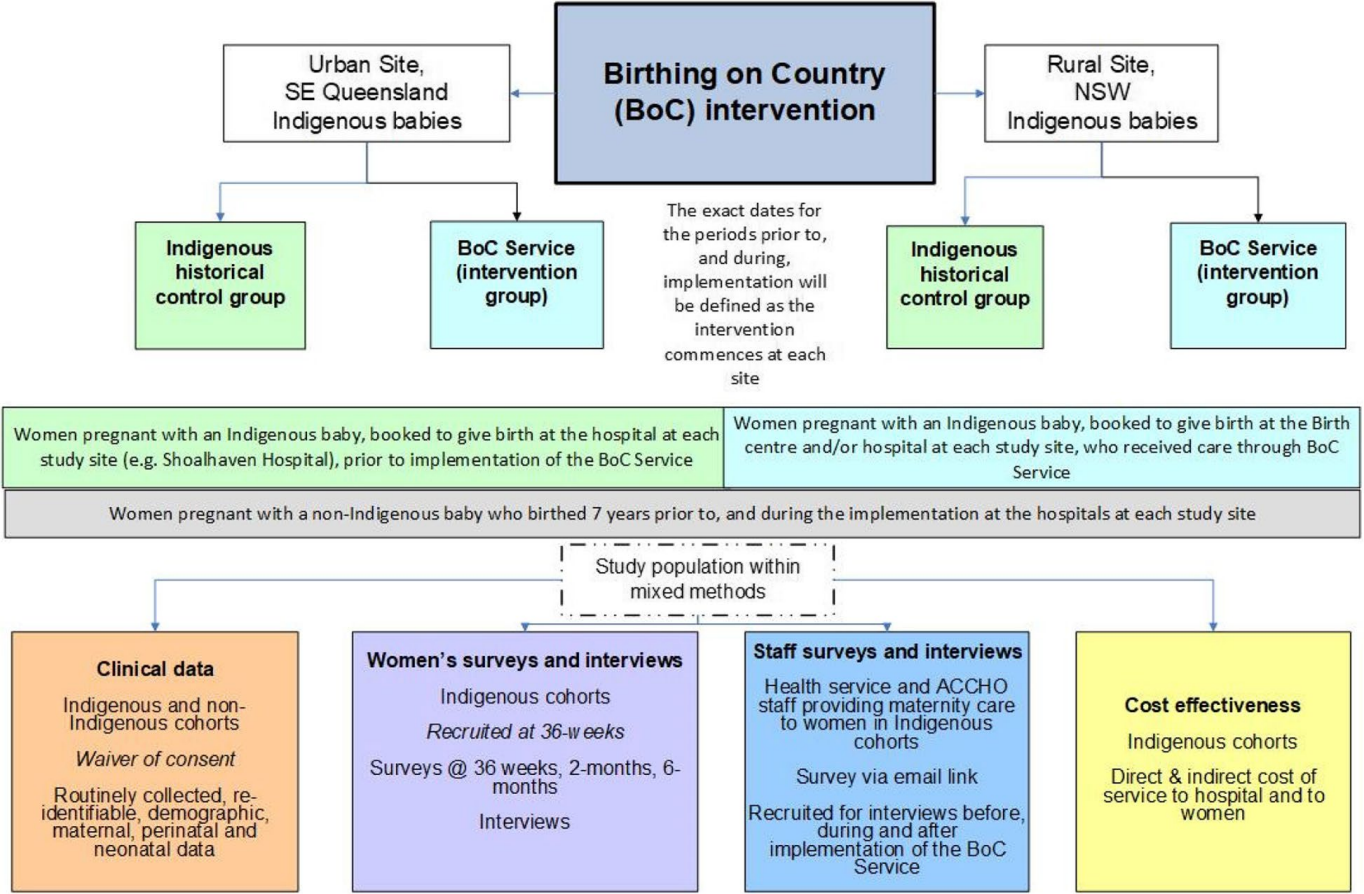
BOOSt study populations and components

The BOOSt study populations are illustrated in Fig. 1. The population groups relevant to clinical outcomes (cohort study), quantitative (surveys) and the qualitative component (interviews, focus groups) are depicted.

### Study components

Using quantitative and qualitative research methods; including clinical and cost-effectiveness data, surveys including questionnaires with validated tools and open response items, interviews, yarning circles and field notes, we will ascertain:

1. Maternal and infant health outcomes (effectiveness): for women obtaining maternity care through *Minga Gudjaga* (Mother & Baby) at Waminda or through BiOC North at IUIH. Routinely collected mother and baby clinical outcomes will enable service effectiveness analysis from commencement of the new service compared with baseline data (standard care) for both sites.
2. Service acceptability: maternal surveys will be undertaken at three time points; approximately 36 weeks antenatally, 2 and 6 months postnatally (see Table 2 & Additional File 2 indicating scales and tools used). A qualitative research component will explore pregnancy, birthing, postnatal and early parenting experiences of a subset of women and will explore cultural safety.
3. Services feasibility, cost-effectiveness and sustainability: Staff and key stakeholder perspectives and experiences will be gathered and will incorporate workforce issues when analysing feasibility and sustainability. A cost-effectiveness analysis of care from the first antenatal visit until 6 weeks after birth will be undertaken by extracting and comparing data on service items, health conditions and diagnoses (therefore procedures and treatments).

**Table 2:** Summary of secondary outcome measures, data collection time points and tools

*#*	*Outcome*	*Method of data collection*	*36 weeks*	*At birth*	*2 months*	*Between 2 & 6 months*	*6 months*
*Cultural safety outcomes*							
1	**Women’s cultural safety views and experiences**	SUR QUAL			X	X	
2	**Women’s experiences of (dis)respect**	SUR QUAL			X	X	
3	**Women’s experiences of discrimination/racism**	SUR QUAL	X		X	X	
*Maternal outcomes during pregnancy, birth and the postnatal period*							
4	**Women’s feelings of control during labour and birth** (rated on a 0–10 scale)	SUR QUAL			X	X	
5	**Smoking status at first antenatal visit** (yes/no)	SUR MEDREC	X (At first AN visit, < 20 weeks, 20 + 1 wks - birth)	X	X		
6	**Experienced continuity of care** measured with 3 subscale scores of the Nijmegen Continuity Questionnaire (NCQ) pregnancy adaptation	SUR QUAL			X	X	
7	**Pharmacological analgesia in labour** (regional [epidural/spinal] and/or narcotic analgesia, nitrous oxide gas)	MEDREC		X			
8	**Onset of labour** (induced, no labour, spontaneous)	MEDREC		X			
9	**Augmentation of labour** with artificial oxytocin	MEDREC		X			
10	**Place of birth** (e.g. home, birth centre, hospital)	MEDREC		X			
11	**Mode of birth** (non-instrumental vaginal birth, instrumental vaginal birth, caesarean section)	MEDREC		X			
12	**Water birth** (yes/no)	MEDREC		X			
13	**Perineal status** (intact/1st degree tear, 2nd degree tear, 3rd/4th degree tear)	MEDREC		X			
14	**Episiotomy** (yes/no)	MEDREC		X			
15	**Management of third stage labour** (active, physiological)	MEDREC		X			
16	**Postpartum hemorrhage** (blood loss < 500; 500–999; 1000–1499; 1500 ml and/or with blood transfusion	MEDREC		X DC – 6 wks			
17	**Mother admission to hospital** up to 6 weeks postpartum (yes/no)	MEDREC		X – 6 wks			
18	**Women who had a known midwife in labour and/or birth** (yes/no)	SUR			X		
19	**Birthing and maternity care experiences**	SUR QUAL	X		X	X	X
20	**Maternal parenting self-efficacy**	SUR QUAL			X	X	
*Fetal, neonatal and infant outcomes*							
21	**Low birth weight** (< 2500 g, 2500 g or more)	MEDREC		X			
22	**Birth weight** (grams, mean, sd)	MEDREC		X			
23	**Apgar score 5 minutes** (< 7, 7 or above)	MEDREC		X			
24	**Admission to a separate neonatal nursery** (yes/no)	MEDREC		X - DC			
25	**Breastfeeding status at 2 and 6 months postpartum** (exclusive, mixed, formula only, other)	SUR			X		X
26	**Infant admission to hospital up to 28 days of age** (yes/no)	MEDREC		X – 28 days			
27	**Length of facility stay for mothers and infants following birth** (mean, median, range)	MEDREC		DC			
*Social & Emotional Wellbeing outcomes/variables, Culture and Connection*							
28	The **Edinburgh Postnatal Depression Scale** (EPDS) score (could also be assessed at other timepoint(s) as clinically indicated)	MEDREC SUR	First AN visit		X		X
29	**Negative Life Events Scale** (NLES) (full extended version) score	SUR	X				X
30	The **modified Kessler psychological distress scale** (K5) score	SUR	X		X		X
31	**Women’s cultural connection and identity**	SUR QUAL	X			X	X
*Acceptability, safety and satisfaction*							
32	The **feasibility and acceptability of an established Birth Centre** assessed by the proportion of eligible women in Birthing on Country care who start labour in the birth centre	MEDREC QUAL		X		X	
33	The **proportion of women who transferred from the birth centre**/model of care at any time and for any reason	MEDREC		X DC			
*Cost outcomes*							
34	**Mean cost of care per mother/infant pair** Health economic evaluation takes a health system perspective and will conduct a cost-effectiveness analysis. Costs to be included are antenatal care, birth, postnatal care costs; readmissions costs within 6 weeks postpartum; infants’ special care nursery and readmission.		First AN visit	Until 6 weeks (mother) 28 days (baby)			

Key: *DC* Discharge (from hospital/birth centre etc), *MEDREC* Medical/maternity/obstetric records, *QUAL* Qualitative data collected via audio recorded, one-to-one yarns (a type of narrative interviewing), unstructured/semi-structured interviews, yarning circles (a type of focus group discussion), researcher field notes, *Sd* Standard deviation, SUR Questionnaires (surveys) designed for this study, *Wk* week

Notes

1. All tools/scales will be scored according to their recommended guidelines and outcomes reported accordingly

2. All items measured for both intervention and standard care groups

3. The time period under consideration for the innovation is from the first day of pregnancy until handover to child health services at 6 weeks postpartum, however the study is longitudinal following mothers and babies until 6 months

4. Please see Additional File 2 for more detail on scales and tools used in women’s surveys

#### Quantitative component

We chose a prospective cohort design to investigate primary and secondary outcomes, incorporating use of standard measures for psycho-social outcomes (e.g. the Edinburgh Depression Scale and Modified Kessler Psychological Distress Scale - K5). Selecting comparison cohorts from the same sites in earlier years for data-based analyses reduces the potential for selection bias. We will describe the general characteristics of women in each cohort to establish comparability.

#### Qualitative component

Multiple qualitative methods and data sources will be used including yarning interviews and yarning circles [50, 51], free text responses in participant surveys, the project log and field diaries/notes, and minutes from meetings. We will also specifically investigate the experiences of women accessing the Waminda Empowering Mothers’ and Babies’ Autonomy (EMBA) process that offers integrated, wraparound multidisciplinary support for First Nations women and families managing complex life circumstances in pregnancy.

#### Cost-effectiveness component

The health economic evaluation will take a health system perspective and conduct a cost-effectiveness analysis from women’s first antenatal visit until 6 weeks after birth. The following costs will be included: antenatal care, birth services, postnatal care, readmissions within 6 weeks postpartum, infants’ special care nursery and readmission within 28 days post birth. The mean cost of care per mother/infant pair will be calculated. The personnel cost including midwife and obstetrician will be estimated using their hourly rate plus on-costs. The costs of diagnostic tests and investigations will be estimated from the government Medicare Benefits Schedule (MBS) and pharmaceuticals from the Pharmaceutical Benefits Schedule (PBS). All hospital admission costs will be based on Australian refined Diagnostic Related Groups (DRGs).

### Outcome measures

#### Primary maternal outcomes

1. First antenatal visit^3^ in the first trimester (< 14 weeks) [for definition please see p. 5 [52]]
2. Total number of antenatal visits (either 0–4, or > =5)
3. Normal birth (> = 37 weeks, vertex presentation, spontaneous onset of labour, no regional analgesia, spontaneous vaginal birth, no episiotomy)

#### Primary infant outcomes

1. Preterm birth (> 20 weeks gestation or 400 g [Australian definition of viability] and < 37 weeks’ gestation)
2. Healthy baby (live born, singleton, > = 37 weeks gestation, 2500–4499 g birthweight, Apgar score at 5 minutes ≥7)
3. Exclusive breast feeding at discharge

#### Secondary outcomes

Secondary outcomes will be assessed in these key areas:

1. Other maternal and infant health and wellbeing status indicators, behaviours and outcomes
2. Services acceptability, effectiveness and cost-effectiveness
3. Services feasibility and sustainability (e.g. number of functioning services, funding attained, indicators of service use, transfers etc).

A full list of secondary outcomes and data sources is summarised in Table 2.

#### Integrated Process Evaluation & Process Outcomes

BOOSt incorporates a comprehensive evaluation protocol to enable monitoring and reporting on what works, for whom, how and why, and in what circumstances. The evaluation will include: a process evaluation to articulate development and implementation, describe contextual factors and mechanisms in operationalising the new Birthing on Country care models; as well as the analysis of feasibility and service sustainability. We consider the following to be critical elements of the innovation and they will be explored in the evaluation: enactment of First Nations governance and the mechanisms supporting it, workforce development; cultural strengthening; family-centred service orientation; and the implementation of the maternity service redesign.

Process outcomes: the components of routine maternity care alongside the proposed key ingredients of a Birthing on Country service are described (Table 1). Related components will be analysed during implementation, including workforce pathways, numbers and types of staff needed and recruited, First Nations recruitment, staff retention, caseload numbers for midwives, health care team cohesion and collaboration etc. First Nations governance indicators will include the functioning of committees, employment by the Aboriginal Community Controlled Health Organisations, functioning of community advisory/engagement groups among others including subjective assessments.

### Study participants & eligibility

#### Inclusion/exclusion criteria

##### Cohort study

Routinely collected data will be extracted for all women within the historical and intervention time periods who gave birth (or were booked to give birth) to a First Nations baby in the study sites (2013–2022). Exclusions will be based on standard and/or previously used methods to ensure validity and rigour.

##### Women’s surveys and interviews

Women aged 14 years and over, pregnant with a First Nations baby and booked for maternity care in either research setting are eligible to participate. Of women in the new care models, we estimate approximately 50% will complete the pregnancy survey. From that group, we estimate based on our previous work, a 70% response rate at 2-months postpartum and a 50% response rate at 6-months postpartum. Of the women recruited to participate in surveys, we estimate approximately 10% will be interviewed.

##### Eligibility

Women are eligible to participate if they:

- Are 14 years and over;
- Are having a First Nations baby;
- Obtained their maternity care through *Minga Gudjaga* (rural Birthing on Country service), or through *Binji & Boori*, or obtained standard maternity care; and are planning to birth in the catchment area;
- Obtained their maternity care through *BiOC North* (urban Birthing on Country service), or obtained standard maternity care; and are planning to birth in the catchment area;
- Provide consent.

##### Health staff surveys and interviews

Health and support workers providing care for families in either setting will be invited to participate in a staff survey and/or interview or focus group discussion. We anticipate from previous experience most MGP midwives within the Birthing on Country service complete surveys and agree to be interviewed. Some hospital staff may participate. Staff are eligible if they have been involved in the planning and/or provision of care including family support, as part of the maternity journey for First Nations families in catchment areas during the study period.

#### Power and sample sizes

BOOSt has been powered to detect a clinically significant change in preterm birth [53]. Preterm birth rates for First Nations babies in the Illawarra Shoalhaven Local Health District have been reported over a recent 5 year period as between 10.3 and 17.5%, average 12.9%. Based on our observations in the IBUS study [19], we propose a minimum treatment effect of 50% reduction (Odds ratio OR 0.5) for the preterm birth rate in the innovation arm. During recruitment, we will aim to access routinely collected maternity data for approximately 687 women at the rural site and 614 from the urban site (based on an estimated 20% attrition rate). From commencement of the new model of care, we will aim to obtain data from a minimum of 296 births (rural site) and 264 (urban). Given small numbers in the rural site (~ 120 births of First Nations babies attending Birthing on Country per year) and a ~ 3.5 year post-intervention study timeframe, we estimate 70% of the total sample to be in the pre-intervention group and 30% post-intervention. To detect this difference with 80% power and a type 1 error of 5%, and factoring exclusions for ~ 7% twin babies/babies with fetal anomaly, and 10% missing data, 983 women/births in total are needed, which includes 687 in pre-intervention and 296 in post-intervention groups. This sample size will also enable enough power for primary infant and maternal outcomes. For the urban site, the required sample size would be 878: 614 in pre-intervention and 264 in post-intervention groups. For the sub-studies, the sample size will be determined according to qualitative research conventions and considerations around data saturation.

### Data collection tools, procedures and ethical considerations

#### Data collection tools

##### Women’s surveys

Survey data will be used to ascertain service acceptability, safety, effectiveness and sustainability. The questionnaires were originally derived from those used in IBUS [54] with the addition of some newer validated tools, however there are few tools that have been validated with Australian First Nations peoples. Additional data items will also be collected based on local factors of importance. Table 2 and Additional File 2 lists the validated tools that are used at each survey time point, the outcome the tool measures, and an approximate time for participant completion. Questionnaires will be pretested and piloted with key stakeholders at the rural site, with subsequent revisions (please see Additional File 2: Scales and Tools used in women’s surveys).

##### Staff surveys

To aid in understanding the new service from a workforce perspective, online anonymous surveys will be conducted with eligible service staff including midwives. Surveys will include questions on relevant topics such as team cohesion, organisational collaboration, cultural capability of staff/team members, and suggestions for improvements. The Copenhagen Burnout Inventory [55] and Perceptions of Empowerment in Midwifery Scale-revised (PEMS-revised) [56] will be used, and these have been tested with Australian midwives providing continuity of care and compared with those working in fragmented models [57]. Additional questions about how the MGP operates (caseload, on-call arrangement etc) will be triggered for participants who work in this service model, and can be explored further in Interviews and/or focus groups. Internal piloting of the survey has been completed.

#### Data collection procedures

##### *Women’s surveys*

Survey data collected at three time points will be used to assess outcomes (including women’s experiences) against study aims. There will be the option of completing surveys independently online or with a researcher. Surveys will include questions related to many aspects of maternity care, as well as general life circumstances (e.g. social, emotional, demographic), and other factors such as cultural identity and connection (see Table 2). Surveys are based on previous research work and include relevant validated instruments that have been used by our team previously (see Additional File 2 for scales and tools to be used).

Piloting and early data collection from the IBUS study identified that most women complete the surveys online, while some were receptive to face-to-face completion with researchers entering data directly into a device or being nearby to answer queries during completion. All jurisdictional restrictions for the COVID-19 pandemic will be followed, and additional precautions will reduce risks to participants and researchers (e.g. mask wearing). Where allowable, options will be offered regarding a meeting place of the participants’ choosing. A $20 grocery/department store gift card will be provided after each survey completion, and a small baby gift will be given following the 2-month survey.

When women complete a survey with a researcher, full verbal explanations will be provided. If a survey response(s) indicates a service issue, with the participant’s permission this may be escalated for further action. Researchers will be aware of local hospitals’ complaints processes, and may help or arrange assistance with this. Where online surveys are completed, and a high score and/or selection of ‘trigger’ responses on particular scales are recorded e.g. the Edinburgh Depression Scale (EDS), designated researchers will receive an immediate alert email. Participants will be offered referral (as per IBUS procedures), and triggers and follow ups will be documented.

##### *Women’s yarns & interviews*

Women will have the opportunity to share their birth, pregnancy and parenting stories in an in-depth narrative or semi-structured interview. Interviews will occur in a convenient and practical location and while face-to-face is preferred, they can be conducted on the telephone or by using digital applications such as Skype© or Zoom©. Researchers will use ‘yarning’, a culturally appropriate conversation that is relaxed, flexible and includes developing relationships with participants prior to and during the story-telling [50, 58]. Women who have expressed interest in participation will be contacted by the research team, and eligible women will be offered participation in either a one-to-one, conjoint interview, or a yarning circle (a culturally appropriate group discussion). All women who participate in surveys will be invited to interview.

Yarns will be women-led with freedom for participants to tell their stories as they wish, and to focus on areas of importance to them. We will use a visual 1-page interview guide, developed from themes identified during prior research in the two study sites [14, 59], and ongoing local consultation. Researchers will aim to explore Indigenous perspectives of culturally safe care (acceptability). We will seek to elicit experiences of various relationships throughout engagement in maternity care, and the impacts of these, including on health behaviours and outcomes (e.g. decision making, breastfeeding, smoking cessation). Researchers will digitally audio record interviews for verbatim transcription, and write field notes including observations and reflections. We estimate the interviews will take up to 1 hour and participants’ time will be compensated with a $40 gift card.

During face-to-face interviews (and yarning circles) usually two researchers will be present. Reasons include ensuring coverage and inclusion of all important elements, and for pragmatic reasons e.g. child minding. Usual consent processes will be followed. Women can have a support person present at an interview (particularly relevant for those with complex challenges/needs). If a participant becomes distressed, the interview will cease, and a referral process will be followed*.* The yarning circle data collection process will utilise the interview guide, however, discussion between participants will be facilitated which may generate more extensive exploration of women’s attitudes, views and experiences [60–62].

We anticipate women will be introduced to the BOOSt study via a verbal explanation (word-of-mouth), website, social media, poster information and/or the study flyer. An invitation to participate with a PICF for interview will be provided for women to consider.

##### *Staff/stakeholder interviews & yarning circles*

All relevant personnel who provide maternity care for women having First Nations babies at both research sites will be invited to participate in interviews or discussion groups, which will use an appropriate interview guide. Other stakeholders of relevance may also be invited, including upon employment exit. Attitudes and perceptions regarding Birthing on Country before, during and after implementation will be elicited. ‘Key ingredients’ of a best practice model of maternity care will be identified, as well as evaluation of acceptability, feasibility, and cost-effectiveness (e.g. scope of practice) of the different care models. Other service aspects will also be explored. Qualitative semi-structured or narrative interviews/discussion groups may last about 1 hour. Focus groups will use the same semi-structured interview guide to encourage discussion, in order to lead to a richer understanding of staff/stakeholder perspectives [60–62].

##### *Routinely collected clinical and costing data*

Routinely collected data for each mother/infant dyad in relevant services will be obtained from several sources, predominantly ordinary records. Relevant permissions will be sought from data custodians. Expenditure data will provide detailed patient-level information on hospital inpatient and primary care service costs. Our partners’ routinely collected services data (e.g. check-up and expenditure data) will be analysed.

##### Young women

This study is not specifically targeting young women, however we will follow guidance included in the National Statement (p. 65) asserting that young women “who are mature enough to understand and consent and are not vulnerable through immaturity in ways that warrant additional consent from a parent or guardian” will be invited to consent in their own right [63]. We will also be guided by the standard practice employed for clinical procedures whereby the best interests of each woman will be assessed on an individual basis. The contributions of young women in the IBUS study were substantial so we have successfully applied the relevant processes.

##### Withdrawal from the study

All PICFs state the participant’s right to withdraw, and reiterate that this would not affect participants’ relationships with staff, their employer, supervisors, colleagues, partner organisations or the researchers, and current or future employment.

#### Participant recruitment, informed consent and waiver

 Documented, informed consent for participation, will be obtained from all participants except where a waiver for the use of routinely clinical data has been approved. The main considerations are participant privacy, wellbeing and safety – including cultural safety. This will be assured by ensuring the relevant Participant Information and Consent Form (PICF) has been understood; adherence to the eligibility criteria; and reminding participants of their rights to decline or withdraw participation without consequence.

##### Women’s surveys

Participation in BOOSt research will be encouraged utilising multiple approaches, including face-to-face, and online dissemination, where we aim for maximum awareness at both study sites. Front line health staff will be engaged in dissemination of information (e.g. flyers), inviting potential eligible participants to submit an ‘Expression of Interest’ (EOI) for researcher contact.

Eligible women will be recruited in the third trimester (approximately 28–36 weeks gestation) to complete surveys. This will involve three surveys: during pregnancy, approximately 2 months and 6 months after birth. Women who have already given birth to a First Nations baby in the past 6 months, are also able to participate, by completing the relevant survey. These women will have the option to retrospectively complete earlier questionnaires pending discussion with a researcher. The limitations, risks and benefits of this approach are recognised.

Following relevant ethics guidelines [64], women will be advised they may defer a participation decision until they have had an opportunity to discuss with a trusted person(s). Any questions will be answered. For participants recruited to complete questionnaires, consent will be acquired for the use of routinely collected electronic health records data, and for merging of records with participants’ survey data. Other service user groups whose de-identified data will be analysed in the study will not be asked for consent, as this research meets conditions stipulated in the National Statement for waiver of consent^4^ [63].

##### ‘My story’: women’s yarns

Women having given birth in the catchment areas will be invited to participate in the qualitative component of BOOSt (‘My Story’). Any person meeting the survey inclusion criteria will be eligible.

##### Service providers, staff & other stakeholder interviews and focus groups

Staff and stakeholders involved in planning, managing, supporting and/or providing maternity care to eligible women in our catchment areas will be eligible to participate in individual or group interviews. Recruitment, consent and interviews will follow processes already outlined, using the Staff/Stakeholder Participant Information and Consent Form. Staff who may be exiting or have exited relevant roles will also be invited. Interview participants may be reimbursed for any costs incurred, although it is expected the interview will occur during usual working hours as part of work activities. These participants will be given a small gift (such as coffee vouchers) to thank them for their time.

##### Staff surveys

All staff closely involved in the new Birthing on Country services will be invited to complete surveys about their experiences in the maternity care models and midwives will be asked to complete additional survey sections due to the significant change involved from the usual ways of working. Invitations will be emailed with an electronic anonymous survey link. Completion and submission of the questionnaire will indicate consent for the use of anonymised data.

##### Waiver

For routinely collected data, we have a waiver of consent approved for all cohorts of women. This covers both clinical and costing outcome measures. Initially the data will be collected in a re-identifiable form so we can merge the data from different sources with each participant given a unique identifier. Once this is completed all identifiable data will be removed prior to analysis.

#### Potential for risk, burdens and benefits to participants

 It is believed that the benefits of participation for First Nations women and families, health workers and others, outweighs the risks. Nevertheless, fatigue, anxiety, fears about confidentiality, potential for feeling coerced and potential emotional or psychological distress are all considered, and risk mitigation and response plans implemented.

### Data management, storage, monitoring and analysis

Our First Nations partners have expressed the importance of data sovereignty, the principles and actions of which will be embedded in project governance. Relevant collaborators will ensure sound data processes such as those related to the validating and synthesis of relevant findings, publication and dissemination including feeding back to participants and other Community members, and all decisions related to data sharing. A data management plan will standardise and guide the management of data throughout the study and all datasets will be stored in a secure institutional Microsoft® Sharepoint storage folder, with restricted access. As this system is backed up regularly, it will be the sole database. Approaches we will take to analysing the quantitative and qualitative data are outlined below. The key benefit of using mixed methods we anticipate, will be better informed decision making and action. Participants’ experiences as service users, providers, or as study partners and stakeholders complement observed quantitative data analysis. Analysis will be conducted by, and/or in collaboration with, First Nations team members.

#### Quantitative data

##### Health services and other routinely collected data

Anonymous data from all women attending the site hospitals and health services during the study period will be extracted and analysed by birth model of care. Quantitative analyses will compare maternal and infant health outcomes between the baseline cohort (both sites) and Birthing on Country service innovation cohort (both sites), as well as standard care/concurrent and historical control groups, and non-First Nations groups (where available).

The clinical and costing outcomes data will be derived from routinely collected electronic healthcare databases at each site. Data will be imported to Stata® or Statistical Package for Social Sciences (SPSS®). While data will initially be re-identifiable (with unique study number) all patient identifiers will be removed. Data cleaning and coding decisions will be documented.

Analysis will be performed with the latest version of the appropriate software and statistical significance will be determined at the 0.05 level. Binary outcome measures will be presented using odds ratios with 95% confidence intervals. Several methods will be used to address selection bias e.g. multivariate logistic regression models, linear regression models, propensity score matching (identifies and controls for confounders e.g. the presence of pregnancy complications), and multilevel modelling controlling for clustering factors. Longitudinal outcomes will be analysed with generalized estimating equations to account for the correlation between observations repeated in the same person. We will use trend analysis to compare rates of preterm birth between First Nations and non-First Nations women, which will demonstrate if this inequity has reduced.

##### Survey data

Survey data will usually be entered at point of collection directly into the Qualtrics® platform by the research participant or researcher. Once recruitment has closed, data will be extracted from Qualtrics® and imported into SPSS® or Stata®. Descriptive analysis will be performed. Additionally, re-identifiable survey data in Stata®/SPSS® (with unique study number) will be merged with clinical data for further analysis. Analysis will compare outcomes from the baseline cohorts and implementation cohorts (both sites). Descriptive statistics will highlight relevant rates, ratios and percentages/proportions.

#### Qualitative and sustainability data

 The research team will manage transfer, transcription and analysis of in-depth interview and yarning circle data. Data collected through digital audio-recorder systems will be saved in the restricted access Sharepoint. Original audio files will then be deleted from devices.

For analysis of the de-identified qualitative data, we anticipate using thematic analysis and culturally-relevant narrative analysis methods as appropriate for yarning [51] and narrative inquiry. Data will be imported and managed in the latest version of NVivo® (QSR International), where a five step process will be undertaken for thematic analysis: (i) immersion in the raw data (ii) identification of a thematic framework (coding scheme) (iii) application and modification of the framework (incorporates revision and refining of coding scheme based on emergence of new themes) (iv) abstraction and synthesis of themes into higher level categories (v) development of association between categories with a view to explaining findings [65]. Any overlap, inconsistencies and outliers in the analysis are to be reconciled; and a final coding scheme confirmed. Standard practice is for interviewees to be assigned a pseudonym and any other uniquely identifying details removed prior to publication. We recognise some of our partners may actually wish to be named [66] which will be offered as appropriate.

Progress reports will be circulated to key investigators as part of the PAR process. Findings will be used iteratively where possible to inform ongoing development of the Birthing on Country services. Steering committees will consider issues or concerns. Investigators and the research team will meet at least annually to provide guidance on study progress. Researcher field notes will be recorded and saved as text files. These documents will be accessed for content and/or thematic analysis.

All data acquired regarding service sustainability will be analysed according to data type. Our team are currently using the RISE Framework [21, 67] and Realist approaches to evaluate the BiOC service (forthcoming).

#### Participant database

 A purpose-built, participant log sheet will be password-protected and secured with restricted access. The file will contain participant contact details for longitudinal follow-up and unique study numbers**.** All paper-based documents will be stored in locked cabinets. Anonymous identifying information will be extracted from each site’s database to enable the creation of a merged mother and infant record using all data sources and a unique study number. Data subsets will also be extracted for blinded analysis.

## Discussion

The BOOSt study will explore the feasibility of establishing two new Birthing on Country services inclusive of a birth centre, in diverse settings, and the processes required to ensure sustainability. BOOSt will investigate clinical effectiveness, cultural safety, costs and cost-effectiveness of these services alongside the acceptability of services for women and families, Communities and health service providers. The BOOSt partnership brings together a unique and multidisciplinary team to enable a novel and strategic approach to health services innovation, which we propose, has the potential to profoundly and positively impact health and wellbeing outcomes for First Nations women, infants and families.

The BOOSt study’s innovative approach draws on evidence from other countries and settings. The partnership will work with First Nations women, Communities and leaders, to make an impact. BOOSt will set a new standard for the provision of First Nations-led and culturally safe maternity care and will offer an evidence-based model for future adaptation. Translating research outcomes requires effective cooperation to enable transfer and take up. ACCHOs will provide the framework for engaging with First Nations women, and will ensure vital knowledge for translating findings for Communities and other interested parties.

## Ethical considerations

This research complies with all relevant national, international and local guidelines and has been approved by Ethics Committees (as listed) including the Aboriginal Health & Medical Research Council (AH&MRC, NSW) Ethics Committee (1448/18). We prioritise respect for the dignity and wellbeing of women and other study participants, and sound project oversight and governance are in place. Relevant procedures have been reproduced and adapted from IBUS.

## Dissemination of results and publications

We will adhere to first principles meaning First Nations voices, lived experiences, interpretations, and considerations will be privileged in all processes. Dissemination activities will occur in consultation and collaboration with our partners.

## Supplementary Information

**Additional file 1.** : BOOSt Study Partner Organisations and their Role(s).docx Table of supplementary information

**Additional file 2.** : Scales & Tools Used in Women’s Surveys.docx Table of Scales and Tools including outcome being measured and estimated completion time (minutes)

## Data Availability

Data sharing not applicable to this article as no datasets were generated or analysed during the current study. The questionnaires/data collection tools may be made available on request and/or may be included with future publications of data/findings.

## Abbreviations

ACCHO: Aboriginal Community Controlled Health Organisation
AH&MRC: Aboriginal Health & Medical Research Council (of NSW)
ANC: Antenatal Care
ATSICHS: Aboriginal and Torres Strait Islander Community Health Service Brisbane Ltd.
BiOC: Birthing in Our Community
BoC: Birthing on Country
BOOSt: Building On Our Strengths
Community/ies: Capital C denotes reference to First Nations Community/ies rather than reference to the community in general
IBUS: Indigenous Birthing in an Urban Setting
IUIH: Institute for Urban Indigenous Health
MGP: Midwifery Group Practice
NHMRC: National Health & Medical Research Council (Australia)
NSW: New South Wales (State Jurisdiction)
PAR: Participatory Action Research
PICF: Participant Information and Consent Form
QLD: Queensland (State Jurisdiction)
SDMH: Shoalhaven District Memorial Hospital

## Footnotes

1 The study is likely to be extended due to disruptions from several natural disasters beginning in December 2019, and the ongoing COVID-19 pandemic (from March 2020).

2 Visiting access agreements means midwives have ‘visiting rights’ to admit women in their care to a government facility in much the same way a specialist medical officer would.

3 An antenatal visit is defined as “a planned visit between a pregnant woman and a midwife or doctor to assess and improve the wellbeing of the mother and baby throughout pregnancy. It does not include visits where the sole purpose is to confirm the pregnancy.”

4 Conditions:

1. Use of routinely collected data which will be extracted and merged using a unique identifier before all other identifying features are removed prior to analysis. This process carries no more than low risk to participants.
2. This research has broad potential benefits for the health and wellbeing of Aboriginal and Torres Strait Islander families which justifies any risk of not seeking consent for access to routinely collected data.
3. It is impracticable to obtain consent.
4. There is no known reason for thinking participants would not have consented had they been asked.
5. There is sufficient protection of privacy and an adequate plan to protect the confidentiality of data
6. The waiver is not prohibited by law.
