# Supplementary material for: Developing and evaluating Birthing on Country services for First Nations Australians: the Building On Our Strengths (BOOSt) prospective mixed methods birth cohort study protocol
Source: BMC Pregnancy Childbirth. 2023 Jan 28;23:77. doi: 10.1186/s12884-022-05277-8 (PMC9883816; doi:10.1186/s12884-022-05277-8)
Supplement: Supplementary file 1 — Additional file 1. : BOOSt Study Partner Organisations and their Role(s).docx Table of supplementary information [file 12884_2022_5277_MOESM1_ESM.docx]

**Additional File 1: BOOSt Study Partner Organisations and their Role(s)**

| **Waminda South Coast Women’s Health and Welfare Aboriginal Corporation** | Key partner at the rural site (NSW) and provides a significant leadership role across all aspects of the project. The birth centre at the rural site will be owned and operated by Waminda. |
| --- | --- |
| **Aboriginal and Torres Strait Islander Community Health Service (ATSICHS) Brisbane Limited** | Key partner at the urban site and will provide leadership across all aspects of the project. |
| **Institute for Urban Indigenous Health (IUIH)** | Key partner at the urban site (QLD) and provides a significant leadership role across all aspects of the project. It is anticipated that the birth centre will be owned and operated by IUIH and/or ATSICHS, Brisbane. |
| **Congress of Aboriginal and Torres Strait Islander Nurses and Midwives (CATSINaM)** | Contributing in-kind funds to assist with the development of the staff training package for culturally safe care. They will provide support and advice as we develop the career pathway from certificate level through to Bachelor education for Aboriginal and Torres Strait Islander midwives. |
| **Australian College of Midwives (ACM)** | Contributing cash and in-kind funds through a philanthropic grant they acquired. They will provide high-level strategic advice regarding midwifery issues and funding for central activities including training. They are providing a lead role in enabling licencing of the Birth Centres in NSW and QLD and in ensuring availability of an appropriate insurance product. |
| **Rhodanthe Lipsett Trust for Indigenous Midwives** (with the Poche Centre for Indigenous Health, University of Sydney) | A demonstration project for a pipeline for the Aboriginal and Torres Strait Islander midwifery workforce which will commence with a Certificate III Health Services Assistant (including Maternal Infant Care skillset component). They will support all sites in capacity building. |
| **Charles Darwin University** | A key partner providing leadership in research and evaluation methodology and methods, and technical expertise for the innovation. They will co-ordinate and chair the research committee meetings, provide central project management, and site project officers/research assistants. The research team will be responsible for ethics and governance, recruitment, data collection, data analysis, and reporting. |
| **University of Sydney** and **University of Queensland** | Partners through the Chief Investigators providing expertise in their particular fields. |
| **My Midwives** | Australia’s largest private midwifery services business providing continuity of maternity care across care settings, in 4 regions. My Midwives has an abundance of critical expertise, directly relevant to the maternity services model under development for BOOSt. |
