# Supplementary material for: Developing and evaluating Birthing on Country services for First Nations Australians: the Building On Our Strengths (BOOSt) prospective mixed methods birth cohort study protocol
Source: BMC Pregnancy Childbirth. 2023 Jan 28;23:77. doi: 10.1186/s12884-022-05277-8 (PMC9883816; doi:10.1186/s12884-022-05277-8)
Supplement: Supplementary file 2 — Additional file 2. : Scales & Tools Used in Women’s Surveys.docx Table of Scales and Tools including outcome being measured and estimated completion time (minutes) [file 12884_2022_5277_MOESM2_ESM.docx]

**Additional File 2: Scales & Tools Used in Women’s Surveys**

| **Outcome being measured** | **Scale or Tool** | **Est. Completion Time**  **(mins)** |
| --- | --- | --- |
| Continuity of care | **Nijmegen Continuity Questionnaire (NCQ) [1] – adapted for childbirth [2]**  The NCQ is a comprehensive, reliable and valid instrument that was developed to measure continuity of care from the patient’s perspective across primary and secondary settings. It has recently been adapted for the childbirth context into a 12-item tool that measures continuity across three areas (3 subscales): care provider knows me, care provider shows commitment, and team/cross boundary continuity. | 5 |
| Stress | Additional worries (IBUS questionnaire) **[3]** | <1 |
| Social support | Four items tangible social support from the 19 item/5 domain RAND **Social Support Survey** tool **[4]** were adapted for the IBUS study questionnaire, where a 3 point Likert was used instead of a 5-point. One additional question incorporating emotional support was added. We replicated that 5 item subscale here for comparison. | 2 |
| Cultural/Community Connectedness, Belonging | Four questions were adapted from the **Longitudinal Study of Indigenous Children (LSIC) (Wave 8) [5] & the National Aboriginal and Torres Strait Islander Health Survey (NATSIHS) (2018-19) [6]:** whether the participant identifies with a Country; is living on Country; whether baby has been on Country, and whether participant feels a sense of belonging in her community of residence. | <1 |
| Experiences of discrimination & racial prejudice | There is a strong rationale for including questions about discrimination in the BOOSt questionnaires. We recognise that Indigenous peoples are disproportionately forced to draw on resilience and coping mechanisms to deal with challenges including psychological distress. Discrimination is recognised as a potential contributor to that distress, including a compounding effect over time. Lived experiences expressed by Community, and a dearth of local data of any sort (inc. prevalence) were also noted, and seven questions were included from the **Growing up in NZ (2009)** **[7]** questionnaire. These ask about perceived recent and lifetime institutional and interpersonal unfair treatment based on race (for both Aboriginal & non-Aboriginal participants). Note: no questions are about other perceived reasons for discrimination, such as pregnancy OR  If participant is Aboriginal, eight questions were included from the **LSIC (Wave 10)** [5] to obtain a measure of the recent frequency with which participants have had to deal with interpersonal racist actions and communications towards Aboriginal people (i.e. over the past 12 months) (based on Williams). If non-Aboriginal, only two of the eight questions were relevant and included. | <5 |
| Culture & Identity | Personal identity - importance of being an Aboriginal &/or Torres Strait Islander. This question has been adapted from the Footprints in Time: **Longitudinal Study of Indigenous Children [5]** (LSIC, variable apl30, Wave 3). | <1 |
| Cultural Connection, Practice & Importance | As we anticipate the ACCHO Birthing On Country model will support cultural connection, six questions were adapted from the Culture & Identity section of the **Growing Up in New Zealand [7], Antenatal Wave** Mother questionnaire. These were tailored slightly differently depending on whether the participant is Aboriginal or non-Aboriginal. | <5 |
| Financial security/insecurity | A set of 5 questions was taken from the **Australian Bureau of Statistics (ABS) NATSIHS 2012/13 [8].** We added one lead in question, as these questions were considered very sensitive, so explanations were believed important to maximise completion. | 5 |
| Life stressors & social issues | **Negative Life Events Scale (NLES)**  The NLES was developed to measure chronic stress in the Aboriginal and Torres Strait Islander population. The Scale generally performed well in a diverse Aboriginal and Torres Strait Islander population sampled during validation and is thought to be a good tool for further research into Social and Emotional Wellbeing in Aboriginal and Torres Strait Islander populations [9]. Our study precursor, the Indigenous Birthing in an Urban Setting (IBUS) study [3] used a full extended version of the NLES as used in a wave of the national survey - NATSIHS (2012-2013, Family Stressors Module 35) [8]. That version has a total of 25 items. We used the IBUS version (including 1 additional item) and added an additional 9 potential stressors. | 5 |
| Social and emotional well-being | **Positive Wellbeing Scale**  Four positively-worded items from the mental health and vitality scales used to measure social and emotional wellbeing in Aboriginal and Torres Strait Islander peoples [10]. (Caveats added as footnotes to ensure relevance for pregnancy & postnatal experience) | 1 |
| Childbirth experience inc. experience of midwife, perceptions of control during labour and birth | **Childbirth Experience Questionnaire (CEQ)**  The Childbirth Experience Questionnaire is a valid and reliable measure of childbirth experience [11] (version translated and validated in English, from Swedish [12]) It uses 22 closed questions (Likert scales and Visual Analogue Scales) across four domains: own capacity, professional support, perceived safety and participation. (2 additional questions added: cultural security and positively worded perception of own capacity. | 5 |
| Parenting self-efficacy | **Adapted Maternal Parenting Self-Efficacy (PMP S-E) tool [13]**  While this tool has been considered a psychometrically robust, reliable and valid measure of parenting self-efficacy, it was designed and validated for use with parents of hospitalized preterm neonates. The original tool has 20 positively-worded phrases and invites women to respond on a 4-point scale from ‘strongly disagree’ to ‘strongly agree’. We accepted the tool is useful for assessing parenting sensitivity but considered 9 statements to be repetitive, not relevant, or inappropriate for our population. We added 3 positively-worded relevant and affirming statements consistent with a strengths-based approach, adapted from the **LSIC PEEM scale** (the Parenting Empowerment and Efficacy Measure) [14]. The total number of questions is 14. | 5 |
| Maternal satisfaction with ANC | **Midwifery Group Practice: women’s satisfaction [15]**  Two open ended questions invited women to list three ‘things you really liked’ and three ‘things you really didn’t like’ about your antenatal care (including MGP care and/or other); coded and analysed thematically. | 5 |
| Sense of respect during healthcare | **Mothers on Respect index (MORi)**  This validated scale includes 14-items, is ideal for measuring quality and safety of maternity care models that prioritise relationship-based care [16]. It can be used to quantify women’s sense of disrespect and dismissal when engaging with providers [16]. | 5 |
| Quality of midwifery care | **Midwife during labour & birth**  A 5 item scale (source unknown) | 1 |
| Cultural safety; felt judged/poor treatment | Cultural safety; felt judged by healthcare staff/experienced poor treatment and perceived reason(s) **modified from the Aboriginal Families Study (AFS) [17]** | 3 |
| Postnatal morbidity | Participants were asked about any postnatal health concerns, as found in the **COSMOS trial [18]**, but with the addition of plain language explanations. | 3 |
| Any perceived discrimination/unfair/inequitable treatment by perinatal healthcare staff | We use **Brown et al’s [19] perinatal adaptation and validated version of the MIRE Measure of Indigenous Racism Experience [20]**. However, we incorporate Likert scale headings adapted from the Menzies Strong Souls (2010) scale [21], a tool used with Aboriginal &/or Torres Strait Islanders that measures social and personal resilience). Note: Brown’s tool elicits recent frequency only (during the previous 12 months), it does not elicit participants’ perceived **reasons** for unfair treatment, only whether they perceived it occurred. Including this tool in the 2 month postnatal questionnaire would capture events occurring during and slightly prior to pregnancy. | 1 |
| Maternity care components, importance & availability | Participants were asked to rate on a 5-point Likert scale, the importance and availability of 13 specific maternity care components. The questions were adapted from an **IBUS study** question asking about satisfaction related to the care components [3]. | <5 |
| Postpartum depressive symptoms | **Edinburgh Postnatal Depression Scale (EPDS)**  The EPDS is a reliable and validated screening tool using 10-items with 4-point responses on a Likert scale [22]. | 3 |
| Psychological distress | **Modified Kessler Psychological Distress Scale**  The 5-item scale has been shown to be a sensitive screen for anxiety and mood disorders; validated for use with Aboriginal and Torres Strait Islander people [10] | 1 |
| Psychosocial stress: impact of major environmental events  Resilience | Given the potential effects of a global pandemic, we included 3 question and adapted an additional cognitive appraisal question from a **Hybrid COVID Stress Scale [23]** using a 7-point Likert, and also adapted that question to capture any effect of widespread summer bushfires (2019-2020).  A moderating question on resilience was included from the **European Social Survey [24]**. | 1 |

**References for Scales & Tools:**

1. Uijen AA, Schellevis FG, van den Bosch WJ, Mokkink HG, van Weel C, Schers HJ: **Nijmegen Continuity Questionnaire: development and testing of a questionnaire that measures continuity of care**. *J Clin Epidemiol* 2011, **64**(12):1391-1399.

2. Perdok H, Verhoeven CJ, van Dillen J, Schuitmaker TJ, Hoogendoorn K, Colli J, Schellevis FG, de Jonge A: **Continuity of care is an important and distinct aspect of childbirth experience: findings of a survey evaluating experienced continuity of care, experienced quality of care and women’s perception of labor**. *BMC Pregnancy and Childbirth* 2018, **18**:13.

3. Hickey S, Roe Y, Gao Y, Nelson C, Carson A, Currie J, Reynolds M, Wilson K, Kruske S, Blackman R *et al*: **The Indigenous Birthing in an Urban Setting study: the IBUS study A prospective birth cohort study comparing different models of care for women having Aboriginal and Torres Strait Islander babies at two major maternity hospitals in urban South East Queensland, Australia**. *BMC Pregnancy and Childbirth* 2018, **18**(1):431.

4. RAND Corporation **Social Support Survey** <https://www.rand.org/health-care/surveys_tools/mos/social-support.html> Date Accessed: 22 November 2022

5. Australian Government **Footprints in Time: The Longitudinal Study of Indigenous Children** <https://www.dss.gov.au/about-the-department/longitudinal-studies/footprints-in-time-lsic-longitudinal-study-of-indigenous-children> Date Accessed: 22 November 2022

6. ABS **National Aboriginal and Torres Strait Islander Health Survey 2018-2019** <https://www.abs.gov.au/statistics/people/aboriginal-and-torres-strait-islander-peoples/national-aboriginal-and-torres-strait-islander-health-survey/latest-release#survey-material> Date Accessed: 22 November 2022

7. Growing Up in New Zealand <https://www.growingup.co.nz/about-growing> Date Accessed: 22 November 2022

8. ABS **National Aboriginal and Torres Strait Islander Health Survey 2012-2013** <https://www.abs.gov.au/ausstats/abs@.nsf/mf/4727.0.55.006> Date Accessed: 22 November 2022

9. Kowal E, Gunthorpe W, Bailie R: **Measuring emotional and social wellbeing in Aboriginal and Torres Strait Islander populations: an analysis of a Negative Life Events Scale**. *International Journal for Equity in Health* 2007, **6**(1):18.

10. Australian Institute of Health and Welfare: **Measuring the social and emotional wellbeing of Aboriginal and Torres Strait Islander peoples.** Canberra: AIHW; 2009.

11. Walker KF, Wilson P, Bugg GJ, Dencker A, Thornton JG: **Childbirth experience questionnaire: validating its use in the United Kingdom**. *BMC Pregnancy Childbirth* 2015, **15**:86.

12. Dencker A, Taft C, Bergqvist L, Lilja H, Berg M: **Childbirth experience questionnaire (CEQ): development and evaluation of a multidimensional instrument**. *BMC Pregnancy Childbirth* 2010, **Dec 10**(10):81.

13. Barnes CR, Adamson-Macedo EN: **Perceived Maternal Parenting Self-Efficacy (PMP S-E) tool: development and validation with mothers of hospitalized preterm neonates**. *Journal of advanced nursing* 2007, **60**(5):550-560.

14. Kikkawa D: **Parenting Efficacy of Parents of Indigenous Children [Data highlight: No.3/2016]**. Canberra: Department of Social Services, Australian Government; 2016.

15. Fereday J, Collins C, Turnbull D, Pincombe J, Oster C: **An evaluation of Midwifery Group Practice Part II: Women's satisfaction**. *Women and Birth* 2009, **22**(1):11-16.

16. Vedam S, Stoll K, Rubashkin N, Martin K, Miller-Vedam Z, Hayes-Klein H, Jolicoeur G: **The Mothers on Respect (MOR) index: measuring quality, safety and human rights in childbirth**. *SSM - Population Health* 2017, **3**:201-210.

17. Buckskin M, Ah Kit J, Glover K, Mitchell A, Miller R, Weetra D, Wiebe J, Yelland JS, Newbury J, Robinson J *et al*: **Aboriginal Families Study: a population-based study keeping community and policy goals in mind right from the start**. *International Journal for Equity in Health* 2013, **12**(41).

18. McLachlan HL, Forster DA, Davey M-A, Lumley J, Farrell T, Oats J, Gold L, Waldenström U, Albers L, Biro MA: **COSMOS: COmparing Standard Maternity care with One-to-one midwifery Support: a randomised controlled trial**. *BMC Pregnancy and Childbirth* 2008, **8**(35).

19. Brown SJ, Gartlanda D, Weetraa D, Leaned C, Francise T, Mitchellf A, Glovera K: **Health care experiences and birth outcomes: Results of an Aboriginal birth cohort**. *Women and Birth* 2019, **32**:404-411.

20. Paradies Y, Cunningham J: **Development and validation of the Measure of Indigenous Racism Experiences (MIRE)**. *International Journal for Equity in Health* 2008, **7**:9.

21. Menzies School of Health Research **Strong Souls Assessment Tool** <https://www.menzies.edu.au/page/Resources/Strong_souls_assessment_tool/> Date Accessed: 22 November 2022

22. Cox JL, Holden JM, Sagovsk R: **Detection of Postnatal Depression: Development of the 10-item Edinburgh Postnatal Depression Scale**. *British Journal of Psychiatry* 1987, **150**:782-786.

23. King S **Hybrid COVID Stress Scale (Version April 8, 2020)** <https://ccna-ccnv.ca/covid-19-useful-resources1/> Date Accessed: 22 November 2022

24. European Social Survey **Round 3** <https://www.europeansocialsurvey.org/data/> Date Accessed: 22 November 2022
